# Clustering of hepatitis C virus antibody positivity within households and communities in Punjab, India

**DOI:** 10.1017/S0950268819001705

**Published:** 2019-10-07

**Authors:** A. Trickey, A. Sood, V. Midha, W. Thompson, C. Vellozzi, S. Shadaker, V. Surlikar, S. Kanchi, P. Vickerman, M. T. May, F. Averhoff

**Affiliations:** 1 Population Health Sciences, University of Bristol, Bristol, UK; 2 National Institute of Health Research (NIHR) Health Protection Research Unit (HPRU) in Evaluation of Interventions at the University of Bristol, Bristol, UK; 3 Dayanand Medical College, Civil lines, Tagore Nagar, Ludhiana, Punjab, India; 4 Centers for Disease Control and Prevention, Atlanta, GA, USA; 5 MSD India Pvt. Ltd, Mumbai, India

**Keywords:** Epidemiology, hepatitis C, serosurvey, statistics

## Abstract

To better understand hepatitis C virus (HCV) epidemiology in Punjab state, India, we estimated the distribution of HCV antibody positivity (anti-HCV+) using a 2013–2014 HCV household seroprevalence survey. Household anti-HCV+ clustering was investigated (a) by individual-level multivariable logistic regression, and (b) comparing the observed frequency of households with multiple anti-HCV+ persons against the expected, simulated frequency assuming anti-HCV+ persons are randomly distributed. Village/ward-level clustering was investigated similarly. We estimated household-level associations between exposures and the number of anti-HCV+ members in a household (*N* = 1593 households) using multivariable ordered logistic regression. Anti-HCV+ prevalence was 3.6% (95% confidence interval 3.0–4.2%). Individual-level regression (*N* = 5543 participants) found an odds ratio of 3.19 (2.25–4.50) for someone being anti-HCV+ if another household member was anti-HCV+. Thirty households surveyed had ⩾2 anti-HCV+ members, whereas 0/1000 (*P* < 0.001) simulations had ⩾30 such households. Excess village-level clustering was evident: 10 villages had ⩾6 anti-HCV+ members, occurring in 31/1000 simulations (*P* = 0.031). The household-level model indicated the number of household members, living in southern Punjab, lower socio-economic score, and a higher proportion having ever used opium/bhuki were associated with a household's number of anti-HCV+ members. Anti-HCV+ clusters within households and villages in Punjab, India. These data should be used to inform screening efforts.

## Introduction

The World Health Organization (WHO) has set global targets for 2030 to reduce new infections of hepatitis C virus (HCV) by 80%, and HCV-related deaths by 65% of the estimated burden in 2015 [1]. In 2015, an estimated 71 million people were living with HCV infection, and 400 000 people die annually of HCV-related complications, mainly end-stage liver disease and liver cancer [2]. Direct-acting antivirals (DAAs) have greatly simplified treatment for HCV infection due to ease of administration (all oral regimens), minimal side-effects and high effectiveness [3]. A better understanding of the risk factors driving historical HCV transmission can support targeted screening and linkage to care, which are needed to reach the WHO targets [4].

The global HCV burden is unevenly distributed; half of all HCV-infected individuals reside in six countries, one being India [5]. India's overall HCV prevalence is an estimated 1%, which is around the global average. However, as India's population is 1.3 billion, the country contains approximately 10 million people living with HCV [6]. Despite a recent systematic review [7], the Indian HCV burden is poorly described because of a paucity of community-level data [7].

India produces the bulk of the world's generic licensed DAAs; therefore, prices are lower than for most countries even after recent price reductions elsewhere [8], reducing an obstacle to treatment access [9]. In 2016, the Indian state of Punjab, which has a population of almost 30 million people [10], launched a programme to provide treatment free of charge [11].

During 2013–2014, a population-based serosurvey was conducted in Punjab finding an overall anti-HCV prevalence of 3.6% and an HCV ribonucleic acid (RNA) prevalence of 2.6% [12]. This serosurvey collected demographic data and information on other exposures possibly associated with HCV [12], identifying associations with the number of blood transfusions received, and the type of practitioner that administered the last medical injection [12]. However, the findings did not account for all possible causes of infection, such as risks associated with having been incarcerated [13]. Additionally, no previous study in Punjab, India, has investigated how HCV is distributed among households.

An understanding of the epidemiology of HCV in Punjab can guide prevention efforts and improve the effectiveness of testing and treatment strategies. The methods outlined for this analysis may be useful for other settings with HCV epidemics that are not concentrated among a specific subgroup of population, such as people who inject drugs (PWID). Understanding how HCV infection is distributed among households or communities can inform screening efforts, resulting in the more effective use of resources, contributing to successful HCV elimination in Punjab. We aim to investigate the distribution and clustering of HCV prevalence within Punjab, India.

## Methods

### Data

This analysis uses data from a cross-sectional seroprevalence survey conducted in Punjab state, India, 2013–2014, described previously [12]. Briefly, the study aimed to estimate HCV antibody (anti-HCV) and viremia prevalence among Punjab residents aged ⩾5 years. The survey included a questionnaire collecting demographic, economic, medical, risk factor and lifestyle information. Participants were tested for anti-HCV and those positive for anti-HCV were tested for HCV RNA by the polymerase-chain reaction. The study employed a multi-stage stratified cluster sampling design, weighted to the 2011 Punjab Census [10]. All persons aged ⩾5 years in a selected household were eligible to participate.

Questionnaires were administered confidentially by trained survey teams via face-to-face interviews for each individual. After each interview's completion, a blood sample was obtained from consenting participants and tested for anti-HCV (Vitros Immunodiagnostic Anti-HCV, Johnson and Johnson Co., New Brunswick, NJ, USA). Those testing positive for anti-HCV are henceforth referred to as anti-HCV+.

The study protocol received approval from the Dayanand Medical College, Ludhiana, Institutional Review Board, and an ethical review committee from the Merck Investigator Initiated Study Protocol-Review Committee. Written consent was obtained for each participant. Confidentiality was strictly adhered to. Participation was voluntary; participants aged 5–17 years provided assent in addition to informed parental/guardian consent.

### Variables considered in regression models

#### Demographic and geographic risk factors

The following characteristics were investigated for association with anti-HCV positivity: age, sex, rural/urban status and north/south residence in Punjab. The districts from the south Punjab included in the study were Muktsar, Moga, Ludhiana, Sangrur and Mansa; the northern districts were Amritsar, Jalandhar, Tam Taran, Gurdaspur and Hoshiarpur. The north/south analysis was included because previous investigations demonstrated prevalences varied by district (Supplementary Fig. S1 [12]); and geographical north/south could be generalised to other provinces not surveyed.

#### Socio-economic status

Previous studies suggest low socio-economic status (S-ES) is associated with increased HCV infection risk from healthcare exposures, such as re-use of syringes [14], which is a common practice in neighbouring Pakistan and has also been identified as a risk factor associated with increased odds of HCV in Punjab, India [15]. A combined, continuous S-ES score variable was created to increase power and better capture S-ES than individual variables.

The following socio-economic indicators were included in the S-ES score variable: household income in rupees (⩽10 000, 10 001–20 000, 20 001–50 000, >50 000; 10 000 rupees is around US$140), whether their residence was a kacha (a flimsy construction) or a pucca (more solid), whether their source of water comes from a tube well, their educational level (none/primary, middle/secondary, graduate) and whether their last healthcare provider used was certified (*vs.* an uncertified/alternative healthcare provider – self-reported).

The S-ES variable was scored on a scale of 0–7 as follows: one point for a household income of 10 001–20 000 rupees and two points for a household income of >20 000 rupees; one point for a pucca residence; one point for not using a tube well for water; one point for completion of middle/secondary school or two points for completing graduate education; one point for receiving healthcare from a certified healthcare provider.

When calculating the probabilities that screening would yield a positive anti-HCV test, we dichotomise the S-ES score into low (⩽3) and high (>3), chosen as the mid-point of the scale.

#### Medical risk factors

Medically-associated risk factors possibly associated with HCV prevalence were: ever had surgery, ever had an invasive medical procedure, ever had a dental procedure, receipt of a medical injection in the previous 6 months, ever received a streptomycin injection for tuberculosis, ever received a blood transfusion and ever been hospitalised.

The combination risk of medical interventions was estimated on a scale scored from 0 to 7, allocating one point for each medical risk the participant had ever been exposed to: surgery, an invasive medical procedure, a dental procedure, a medical injection in the last 6 months, a streptomycin injection, a blood transfusion, hospitalisation.

#### Social and other risk factors

Social risk factors (ever had a tattoo, shaving by a barber (as opposed to at home), ever had a body piercing) and other risk factors (ever been incarcerated and ever had a motor accident) could also be associated with HCV prevalence.

Injection drug use (IDU) is considered a driver of HCV transmission in Punjab [16]. PWID have a high HCV prevalence [17]. However, only five subjects (0.1%) surveyed admitted to having ever injected drugs, a percentage similar to the estimated prevalence of current IDU in Punjab [18]. The prevalence of those currently injecting drugs should be much lower than of ever injecting. Our survey proportion of ever injectors likely represents an underestimate of the actual prevalence. A report from 2008 linked smoking traditional, plant-based drugs to IDU [19]. We examined other drug exposures/behaviours to investigate using them as proxy measures of IDU. The exposures included: ever used opium or bhuki (an intoxicating wild grass that is ingested [20]), ever drank alcohol and ever smoked tobacco.

### Clustering of anti-HCV+ prevalence by household and ward/village

#### Individual-level analyses

For individual-level analyses, study subjects were stratified by urban/rural residence, defined by the 2011 Punjab Census [10], and weighted by population sizes of the wards (areas within cities) and villages and clustered by household. Logistic regression was used to estimate the associations between S-ES score and anti-HCV status, and medical risk score and anti-HCV status, both overall and stratified by rural/urban setting.

An individual-level logistic regression was also used to estimate odds ratios (ORs) and adjusted odds ratios (aORs) for anti-HCV positivity by various characteristics and risk factors, including a variable of whether another household member was positive for HCV antibodies. This analysis was repeated with HCV RNA positivity as the endpoint.

#### Simulation analyses

To further investigate whether anti-HCV+ persons clustered within households, the observed frequencies of households containing multiple anti-HCV+ members were compared with the expected number from simulated data. This simulation assumed anti-HCV+ persons were randomly distributed with a Binomial distribution with a mean equal to the proportion of anti-HCV+ cases in the unweighted survey data. Using the same household structure as found in the survey, 1000 simulations were performed, accounting for the varying urban/rural prevalences. We assumed the number of household members surveyed was a proxy for the actual number of individuals living in the household. This simulation method was repeated for analyses investigating clustering of anti-HCV+ persons within the village/ward level.

#### Household-level analyses

Household characteristics were tabulated by the number of anti-HCV+ members (0, 1 and ⩾2). Ordered logistic regression models were used to estimate associations between each exposure/characteristic and the number of anti-HCV+ members in a household (0, 1 or ⩾2), adjusting for the number of household members, as households with more members have a greater probability of containing anti-HCV+ household member(s). The exposure/characteristics that were associated (*P* < 0.05) with anti-HCV+ household members (when only adjusted for the number of household members) were then included in an ordered logistic regression backwards elimination model. The S-ES score variable, rather than its individual components (e.g. household income), was included in the backwards elimination model, to increase power. A likelihood-ratio test of proportionality of odds across response categories was performed to test the multivariable ordered logistic regression's assumption of proportional odds.

## Results

There were 5543 eligible participants with available HCV testing results who completed the survey, described previously [12]. Briefly, 62% of the surveyed population lived in rural areas, 54% were women and the median age was 35 years (interquartile range (IQR): 21–50). The overall anti-HCV prevalence was 3.6% (95% confidence interval (95% CI) 3.0–4.2%) and was higher in rural areas, 4.7% (95% CI 3.8–5.7%), than urban areas, 1.6% (95% CI 1.1–2.2%). Anti-HCV prevalence varied by district (Supplementary Fig. S1) and was higher in the south (4.7% (95% CI 4.0–5.5%)) than the north (2.0% (95% CI 1.4–2.6%)).

The number of members surveyed in each of the 1593 households was 1, 2, 3, 4 and ⩾5 in 257 (16.1%), 315 (19.8%), 343 (21.5%), 296 (18.6%) and 382 (24.0%), respectively. The largest household had 21 participants surveyed. The median number of household members was 3 (IQR: 2–4). There were 1433 (90.0%) households that had no members who tested anti-HCV+, 130 (8.2%) had one person who tested anti-HCV+, and 30 (1.8%) households had ⩾2 test anti-HCV+. The greatest number of persons testing anti-HCV+ in a household was 4.

### Individual-level analyses

**Fig. 1.**
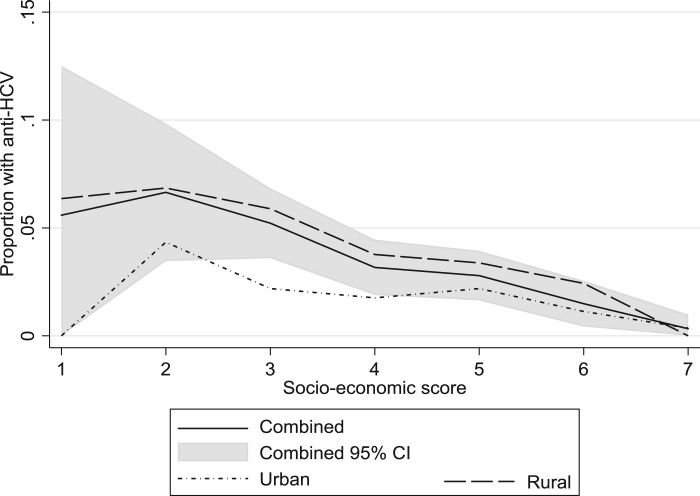
The proportion of hepatitis C virus antibody (anti-HCV) positive individuals by socio-economic score (higher score is more affluent), for all participants (with 95% confidence interval), urban participants and rural participants.

**Fig. 2.**
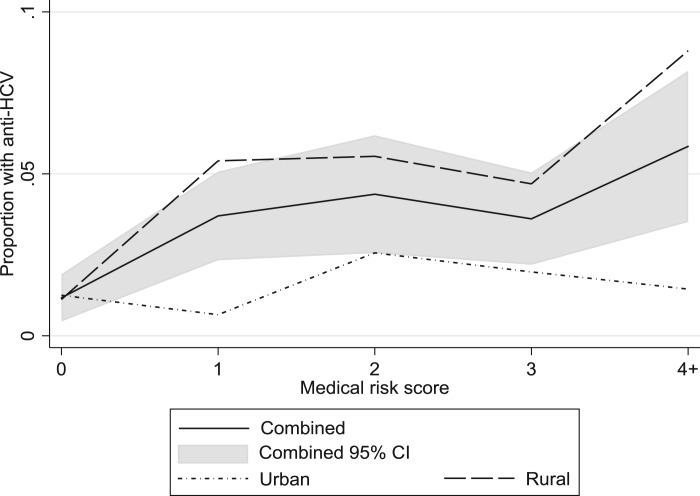
The proportion of hepatitis C virus antibody (anti-HCV) positive individuals by medical risk score, for all participants (with 95% confidence interval), urban participants and rural participants.

In individual-level analyses, the proportion of anti-HCV+ people decreased with an increasing socio-economic score (OR 0.69 (95% CI 0.62–0.77)) (Fig. 1 and Table 1). This effect persisted among both rural residents (OR 0.76 (95% CI 0.66–0.88)) and urban residents (OR 0.76 (95% CI 0.61–0.95)) (Fig. 1). The combined number of medical exposures was positively associated with anti-HCV prevalence (continuous OR per additional medical exposure 1.31 (95% CI 1.17–1.46)) (Fig. 2). The effect was stronger for rural than urban residents: ORs 1.37 (95% CI 1.21–1.55) and 1.16 (95% CI 0.92–1.46), respectively.

**Table 1.** Logistic regression odds ratios (95% confidence intervals) of hepatitis C virus antibody positivity by individual characteristics (*N* = 5543 individuals)

	Odds ratio (95% CI) for having anti-HCV	
Variable	Unadjusted	Adjusted^a^
Another member of household anti-HCV+	4.53 (3.28–6.27)	3.19 (2.25–4.50)
Living in rural dwelling	3.01 (2.07–4.39)	1.57 (1.02–2.42)
Living in the south	3.25 (2.23–4.73)	2.60 (1.75–3.87)
Age (years)	1.03 (1.02–1.03)	1.02 (1.01–1.03)
Male	1.28 (0.94–1.73)	
Medical risks		
Ever had surgery	1.54 (1.14–2.09)	
Ever had a medical procedure	2.20 (1.23–3.92)	
Ever had a dental procedure	1.62 (1.20–2.19)	
Had a medical injection in the last 6 months	1.48 (1.09–2.01)	
Ever had a streptomycin injection	2.40 (1.01–5.72)	
Ever received blood	1.96 (1.21–3.18)	
Ever been hospitalised	1.43 (1.05–1.95)	
Medical risk score^a^	1.31 (1.18–1.46)	1.17 (1.02–1.33)^a^
Socio-economic indicators		
Receiving water through a tube well	2.25 (1.66–3.04)	
Certified healthcare	0.53 (0.37–0.75)	
Kacha (less solid) *vs.* pucca house (more solid)	1.32 (0.83–2.10)	
Household income (rupees)		
0–10 000	1	
10 001–20 000	0.74 (0.52–1.05)	
>2000	0.58 (0.36–0.92)	
Education level		
None/primary	1	
Middle/secondary	0.70 (0.51–0.96)	
Graduate	0.21 (0.10–0.46)	
Socio-economic status score^a^	0.69 (0.62–0.77)	0.75 (0.66–0.86)^a^
Drugs (ever taken)		
Ever drank alcohol	0.62 (0.44–0.88)	0.56 (0.38–0.84)
Ever used opium/bhuki	5.06 (3.25–7.90)	2.85 (1.71–4.76)
Ever smoked tobacco	1.21 (0.63–2.32)	
Social risks		
Have a tattoo	1.54 (0.98–2.44)	
Use barbers	1.52 (1.08–2.14)	1.78 (1.22–2.60)
Have body piercings	0.84 (0.53–2.85)	
Other variables		
Ever been incarcerated	2.70 (1.20–6.08)	1.42 (0.58–3.46)
Ever had a motor vehicle accident	1.77 (1.25–2.48)	1.33 (0.92–1.95)

a For power only the combined socio-economic status score variable was included rather than the socio-economic variables, and similarly only the combined medical risk score from the individual medical risk variables. All other variables that were associated with anti-HCV in the single variable analysis were then included in the multivariable analysis.

Table 1 shows the aOR of a household member being anti-HCV+ if another member of that household is anti-HCV+ 3.19 (95% CI 2.25–4.50). Living in a rural dwelling was also positively associated with being anti-HCV+ (aOR 1.57 (95% CI 1.02–2.42)), as was living in the south (aOR 2.60 (95% CI 1.75–3.87)), age (aOR 1.02 (95% CI 1.01–1.03)), having ever used opium/bhuki (aOR 2.85 (95% CI 1.71–4.76)) and being shaved by a barber (aOR 1.78 (95% CI 1.22–2.60)). Ever having drank alcohol was negatively associated with being anti-HCV-positive, aOR 0.56 (95% CI 0.38–0.84).

Supplementary Table S1 gives a similar analysis to that presented in Table 1 but with HCV RNA positivity as the endpoint. The results of this analysis are similar to those in Table 1, except shaving at a barber (OR 1.45 (95% CI 0.97–2.18)) and having ever been incarcerated (OR 2.54 (95% CI 0.97–6.63)) were not associated with HCV RNA in single variable analyses. In multivariable analyses having ever had a motor vehicle accident (aOR 1.61 (95% CI 1.04–2.50)) was positively associated with HCV RNA positivity, which was not the case in the analysis with anti-HCV positivity as the outcome. The association between being HCV RNA-positive and another member of the household being HCV RNA-positive was very strong (aOR 3.88 (95% CI 2.56–5.89)), as was the association with socio-economic status score (aOR 0.67 (95% CI 0.57–0.78)), and the associations with living in the south (aOR 2.64 (95% CI 1.64–4.26)) and having ever used opium/bhuki (aOR 3.02 (95% CI 1.67–5.48)).

### Simulations

**Fig. 3.**
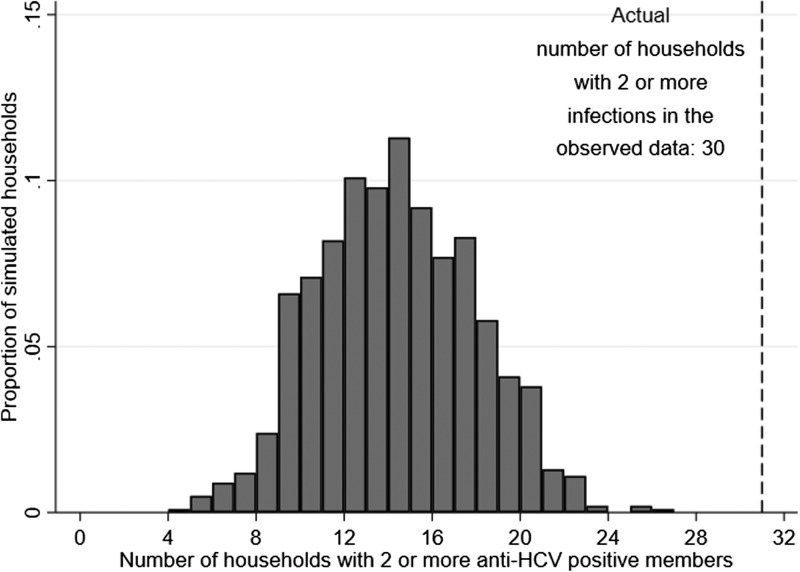
The distribution of the number of households with two or more hepatitis C virus antibody (anti-HCV) positive members in the 1000 simulated datasets assuming HCV randomly distributed, compared to the observed number of households with two or more members with HCV (the dashed line).

Using the same household size distribution found in the survey, 1000 simulations were conducted, with anti-HCV+ persons randomly distributed among households. The simulations resulted in a median of 14 (IQR: 11–16) households with ⩾2 members testing anti-HCV+. Figures 3 shows that none of the 1000 simulations had 30 households with ⩾2 anti-HCV+ members (*P* < 0.001).

**Fig. 4.**
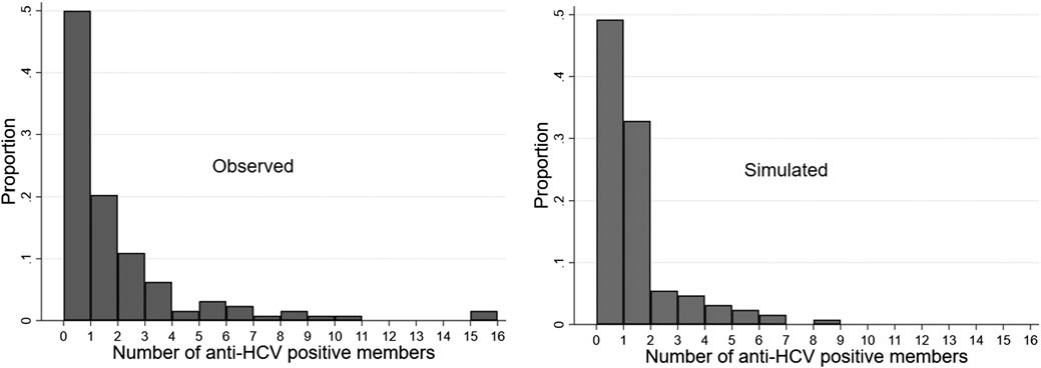
Histograms showing the number of hepatitis C virus antibody (anti-HCV) positive members of each village/ward (left panel: observed, right panel: average of 1000 simulations).

We compared the distribution of the number of anti-HCV+ persons from each village from the survey with the average number of persons testing positive across 1000 random simulations. We found a greater proportion of villages surveyed had multiple persons anti-HCV+ than in the simulations. For example, 31 of the 1000 simulated datasets had 10 villages with ⩾6 infections, the number observed in the survey data (*P* = 0.03; Fig. 4).

### Household-level analyses

As household size increased, the number of members in the household testing anti-HCV+ increased (Table 2). The number of members testing anti-HCV+ in the household was also associated with living in rural areas and living in Punjab's south. Households with a higher proportion of members that had received a medical injection in the last 6 months were more likely to have more anti-HCV+ members. Select indicators of lower S-ES, including receiving water from a tube well, lower educational level and receiving healthcare from an uncertified/alternative provider, were associated with a greater number of household members testing anti-HCV+, as was a lower S-ES score. The proportion of members of the household that had ever used opium or bhuki was also associated with having more anti-HCV+ members.

**Table 2.** Comparing the mean characteristics of households (*N* = 1593) with 0, 1 and ⩾2 members testing positive for hepatitis C virus antibody, respectively

	Number of members anti-HCV^a^ positive in a household			
	0	1	2+	
	Number of households			Test for differences^b^
Characteristic	1433	130	30	
Mean number of members in the household	3.32	4.72	5.67	*P* < 0.001
Mean proportion in rural dwellings	0.60	0.79	0.87	*P* < 0.001
Mean proportion living in the south	0.56	0.72	0.90	*P* < 0.001
Mean age	38	37	40	*P* = 0.441
Mean proportion male	0.45	0.48	0.51	*P* = 0.320
Medical risks				
Mean proportion that have had surgery	0.44	0.41	0.49	*P* = 0.497
Mean proportion that have a medical procedure	0.04	0.03	0.05	*P* = 0.620
Mean proportion that have had a dental procedure	0.41	0.40	0.47	*P* = 0.571
Mean proportion that have had a medical injection in the last 6 months	0.34	0.41	0.39	*P* = 0.049
Mean proportion that have had a streptomycin injection	0.01	0.02	0.01	*P* = 0.578
Mean proportion that have received blood	0.07	0.09	0.07	*P* = 0.537
Mean proportion that have been hospitalised	0.55	0.54	0.58	*P* = 0.795
Mean medical risk score	1.9	1.9	2.1	*P* = 0.479
Socio-economic indicators				
Mean proportion receiving water from a tube well	0.32	0.45	0.68	*P* < 0.001
Mean proportion with certified healthcare^c^	0.40	0.27	0.23	*P* < 0.001
Mean proportion in a kacha house (less solid structure)	0.10	0.17	0.04	*P* = 0.022
Household income^d^	0.62	0.50	0.47	*P* = 0.133
Education level^d^	1.76	1.64	1.63	*P* = 0.015
Mean socio-economic status score	4.2	3.7	3.5	*P* < 0.001
Drugs (ever taken)				
Mean proportion that have ever drunk alcohol	0.33	0.34	0.30	*P* = 0.827
Mean proportion that have ever used opium/bhuki^e^	0.03	0.07	0.16	*P* < 0.001
Mean proportion that have ever smoked tobacco	0.05	0.03	0.05	*P* = 0.257
Social risks				
Mean proportion with tattoo	0.09	0.10	0.08	*P* = 0.588
Mean proportion using barber	0.19	0.21	0.25	*P* = 0.374
Mean proportion with body piercings	0.56	0.54	0.49	*P* = 0.370
Other variables				
Mean proportion that have ever been incarcerated	0.01	0.02	0.03	*P* = 0.324
Mean proportion that have ever had a motor vehicle accident	0.19	0.20	0.27	*P* = 0.286

a Hepatitis C antibody-positive.

b ANOVA test (single variable).

c Receiving healthcare from a certified healthcare provider (as opposed to an uncertified/alternative health care provider).

d Education and household income are using an ordinal variable where lower categories indicate lower education or income.

e Bhuki is an intoxicating wild grass that is ingested.

The associations between household-level variables and the number of anti-HCV+ household members (0, 1 or ⩾2) are shown in Table 3. In models only adjusted for the number of household members, the variables associated with a greater number of anti-HCV+ members in the household were: living in a rural area, living in south Punjab, a higher proportion of the household having had a medical injection in the last 6 months, receiving water through a tube well, a higher proportion having ever taken opium/bhuki and a higher proportion having ever been incarcerated. Conversely, a higher household income, education level, proportion of the household with certified healthcare and a higher mean S-ES were all associated with a fewer number of members in the household testing anti-HCV+. A model adjusted for multiple variables found that several factors were independently associated with an increase in the number of household members testing anti-HCV+: more members living in a household, living in south Punjab, a lower mean S-ES and a higher proportion of household members having ever used opium or bhuki. The proportionality of odds assumption test did not find strong evidence against this assumption (*P* = 0.093).

**Table 3.** Multivariable ordered logistic regression odds ratios (95% confidence intervals) of hepatitis C virus antibody positivity by household characteristics (*N* = 1593 households)

	Odds ratio (95% CI) for having 0, 1 or ⩾2 members in the household with anti-HCV^e^	
	Adjusted^a^	Fully adjusted, backwards elimination^b^
Number of members in the household	1.31 (1.22–1.40)^c^	1.38 (1.28–1.48)
Living in rural dwellings	2.88 (1.87–4.43)^c^	
Living in the south	3.29 (2.14–5.04)^c^	3.15 (1.98–5.02)
Mean age	1.01 (1.00–1.02)	
Proportion male	1.83 (0.90–3.72)	
Medical risks		
Proportion that have had surgery	0.87 (0.47–1.61)	
Proportion that have had a medical procedure	1.05 (0.30–3.66)	
Proportion that have had a dental procedure	1.13 (0.67–1.91)	
Proportion that have had a medical injection in the last 6 months	2.25 (1.30–3.88)^c^	
Proportion that have had a streptomycin injection	1.50 (0.20–11.11)	
Proportion that have received blood	2.21 (0.82–5.93)	
Proportion that have been hospitalised	1.04 (0.60–1.80)	
Mean medical risk score	1.16 (0.95–1.43)	
Socio-economic indicators		
Receiving water through a tube well	1.94 (1.33–2.84)	
Proportion with a certified healthcare provider (*vs*. uncertified/alternative)	0.34 (0.20–0.58)	
Kacha (less solid) vs pucca house (more solid)	1.77 (1.05–2.98)	
Household income^d^	0.64 (0.49–0.85)	
Education level^d^	0.51 (0.36–0.74)	
Mean socio-economic status	0.61 (0.51–0.74)^c^	0.63 (0.55–0.74)
Drugs (ever taken)		
Proportion that have ever drank alcohol	0.66 (0.35–1.24)	
Proportion that have ever used opium/bhuki	26.68 (9.05–78.62)^c^	16.69 (5.46–51.02)
Proportion that have ever smoked tobacco	0.36 (0.06–2.16)	
Social risks		
Proportion with a tattoo	1.67 (0.63–4.44)	
Proportion using barbers	1.46 (0.69–3.07)	
Proportion with body piercings	0.60 (0.31–1.17)	
Other variables		
Proportion that have ever been incarcerated	6.01 (1.11–32.59)^c^	
Proportion that have ever had a motor vehicle accident	1.74 (0.89–3.39)	

a Adjusted for the number of members in the household.

b Variables that were associated with HCV in the analyses only adjusting for number of members in the household (the second column), denoted c, were entered into a backwards elimination model, with the third column presenting the variables that were selected by the backwards elimination model (for power only the mean socio-economic status was included in the selection model rather than the individual socio-economic variables).

c Variables entered into the backwards elimination model.

d Education and household income are using ordinal variables where lower categories indicate lower education or income.

e Hepatitis C antibodies.

### Screening probabilities

The survey results translate to 13.0% (*n* = 120) of 926 households surveyed in Punjab's south containing someone anti-HCV+ and 2.9% (*n* = 27) of these having ⩾2 anti-HCV+ members. These numbers increase to 19.1% and 4.4%, respectively, for the 230 households surveyed in the south that also had a lower S-ES score (⩽3). For the north, 6.0% of the 667 households surveyed contained someone anti-HCV+, which increased to 9.2% when limited to the 164 households with a lower S-ES score. Of the 194 households surveyed with ⩾1 member that uses either bhuki or opium, 26.3% had one anti-HCV+ member and 9.8% had ⩾2 anti-HCV+ members.

## Discussion

In Punjab, India, anti-HCV+ individuals cluster within households and within villages, with higher prevalence in the south than the north. The number of anti-HCV+ household members was positively associated with the number of household residents, lower S-ES and greater use of opium or bhuki in these households. In single variable analyses only adjusted for the number of household members, anti-HCV status was positively associated with the average number of medical injections received in the last 6 months, the proportion of the household that had been incarcerated, as well as socio-economic markers such as using an uncertified healthcare provider, or receiving water from a tube well. These findings help to elucidate HCV infection in Punjab and could guide prevention and screening strategies for the state-wide care and treatment programme [11].

HCV infection is not evenly distributed geographically [7]. Reasons for south Punjab's higher HCV prevalence are uncertain but could be due to poorer infection control practices, more syringe re-use or unreported IDU.

Taking opium or bhuki should not transmit HCV because they are not injected. However, they were strongly related with anti-HCV positivity, possibly indicating they are proxy markers for IDU. Stigma may cause under-reporting of IDU, with the proportion reporting ever injecting drugs lower than recently estimated in Punjab [18]. PWID may be under-represented in household surveys because they are more likely to be homeless or imprisoned [13, 21]. Although some evidence suggests opium/bhuki use could be associated with heroin use [19], its use as a marker of IDU needs further study to understand the validity of using such proxies.

Household anti-HCV positivity was inversely associated with the household's S-ES. Lower-income households may lack access to higher quality healthcare, leading to a greater risk of iatrogenic HCV transmission.

### Literature comparison

Anti-HCV positivity clustering within households could be due to intra-familial transmission between household members or by household members being exposed to common risks outside the house, such as sharing a doctor [22]. Our study cannot determine if households with multiple persons testing anti-HCV+ acquired their infection from exposures within or outside the household [23]. Other studies have looked at HCV clustering at the household/family level, with some in low prevalence countries finding an association [24, 25] or not [26–28], while others in higher prevalence countries [5] found associations. One study found intraspousal HCV transmission more common than other intrafamilial transmission [29]. The association found between low S-ES and anti-HCV positivity has been demonstrated previously, including in Thailand [30], the Netherlands [31] and Pakistan [32, 33]. Utilizing advanced molecular diagnostics, such as deep-gene sequencing, could better define transmission patterns within households, elucidating risks and guiding prevention efforts.

### Strengths and limitations

This study analysed data from a large serosurvey, covering diverse areas of Punjab. However, the sampling frame used census data, which may underestimate the state's anti-HCV prevalence as it excludes homeless populations, new arrivals, prisons and new peri-urban slums. We were unable to correct the overall prevalence estimate to account for the prevalence among these populations due to a lack of data. A cross-sectional serosurvey asking about recent behaviours cannot accurately capture the effect of lifetime medical exposures and injections with contaminated needles, which are important factors associated with HCV prevalence in India, South Asia and globally [15, 34]. The receipt of medical injections is very common in this population, with around 35% of the sample having received one in the previous 6 months. This cross-sectional serosurvey is limited to identifying behaviours associated with prevalent, rather than recent infections, and was likely subject to recall and social desirability bias, particularly affecting the reported prevalence of ever having injected drugs. The proxy measures used possibly captured an effect other than IDU such as low socio-economic status, which is itself a proxy measure, possibly of utilizing unsafe healthcare providers [14]. The negative association between having ever drank alcohol and being anti-HCV-positive is probably a proxy measure for S-ES, maybe caste or religion [35]. This study can only estimate associations, which may be subject to unmeasured confounding. The simulation analyses may be limited by high heterogeneity in anti-HCV prevalence between districts. We were unable to further stratify the household analyses by the number of anti-HCV+ members in each household due to a lack of households containing ⩾3 multiple infected persons. Treatment for HCV in Punjab before this survey was administered was rare and would have been unlikely to affect the anti-HCV or RNA prevalence in the study population.

### Implications

This study found anti-HCV+ persons clustered in households and in villages in Punjab, India. This is an important consideration for the recently launched treatment programme in Punjab aiming to eliminate hepatitis C [1, 15]. Officials should consider testing whole families when one family member tests positive for anti-HCV or HCV RNA. This may achieve a higher yield than general testing. Similarly, the reasons for clustering of anti-HCV+ persons in villages could be from sharing a healthcare provider, or barber or high IDU prevalences in some villages. Further research is required to understand why infection clusters at the village and household levels. In households, there is a range of possible factors, including sexual transmission, sharing razors or using the same barber or healthcare provider with poor infection control practices [36].

Understanding these factors will help planners implement interventions that could prevent HCV transmission in this context and are valuable for initiatives linking those infected to care. For those designing hepatitis C testing and prevention programmes in Punjab, this study provides valuable information, including that households with lower S-ES and households in the south tend to have more anti-HCV+ members. This indicates these groups may benefit from targeted testing and treatment. Furthermore, in Punjab's south, there is approximately a 13% chance that any household screened will yield someone anti-HCV+. This probability increases to roughly 17% for poorer households. Households that have a member reporting opium or bhuki use have over 25% chance of a household member having HCV infection. Information on HCV transmission risks and how to sterilise medical equipment should be targeted to medical practitioners, particularly in high prevalence areas. This could reduce HCV transmission in Punjab, which combined with scaled-up treatment should reduce the high HCV prevalence.

## Supplementary material

For supplementary material accompanying this paper visit https://doi.org/10.1017/S0950268819001705. click here to view supplementary material
