# Supplementary material for: Clustering of hepatitis C virus antibody positivity within households and communities in Punjab, India
Source: Epidemiol Infect. 2019 Oct 7;147:e283. doi: 10.1017/S0950268819001705 (PMC6805795; doi:10.1017/S0950268819001705)
Supplement: Supplementary file 1 [file S0950268819001705sup001.docx]

**Clustering of hepatitis C virus antibody positivity within households and communities in Punjab, India**

**Supplementary materials**

**Supplementary figure 1:** The prevalence of hepatitis C (anti-HCV) for each district that participated in the survey, re-printed with permission from Sood et al, 2018*(1)*.


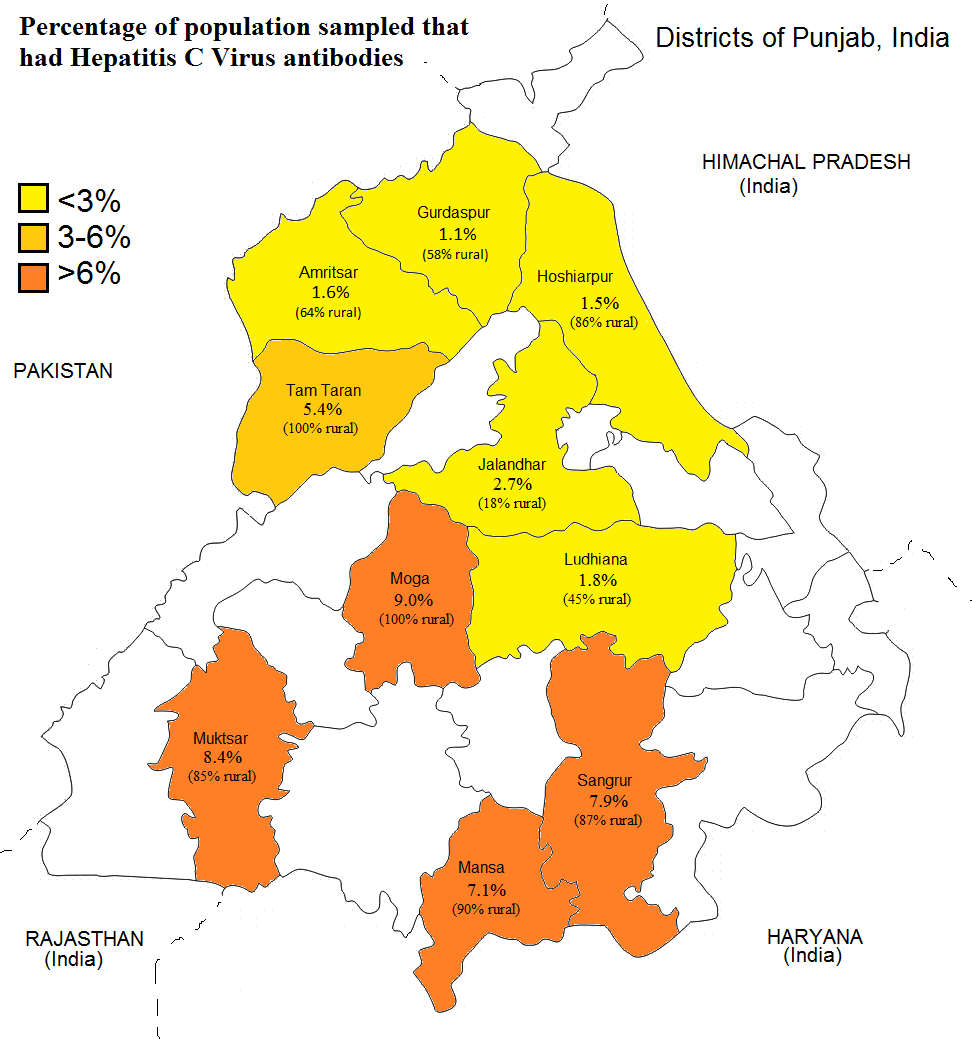


**Supplementary table 1:** Logistic regression odds ratios (95% confidence intervals) of hepatitis C virus RNA by individual characteristics (N=5543 individuals)

|  | Odds Ratio (95% CI) for having HCV RNA | |
| --- | --- | --- |
| Variable | Unadjusted | Adjusted* |
| Another member of household HCV RNA+ | 6.11 (4.13, 9.04) | 3.88 (2.56, 5.89) |
| Living in rural dwelling | 3.57 (2.24, 5.69) | 1.54 (0.91, 2.60) |
| Living in the south | 3.30 (2.10, 5.20) | 2.64 (1.64, 4.26) |
| Age (years) | 1.02 (1.02, 1.03) | 1.01 (1.00, 1.02) |
| Male | 1.23 (0.86, 1.77) |  |
| *Medical risks* |  |  |
| Ever had surgery | 1.66 (1.16, 2.38) |  |
| Ever had a medical procedure | 2.33 (1.18, 4.57) |  |
| Ever had a dental procedure | 1.74 (1.22, 2.50) |  |
| Had a medical injection in the last 6 months | 1.42 (0.99, 2.05) |  |
| Ever had a streptomycin injection | 0.77 (0.23, 2.62) |  |
| Ever received blood | 2.22 (1.28, 3.83) |  |
| Ever been hospitalized | 1.47 (1.02, 2.13) |  |
| Medical risk score* | 1.33 (1.18, 1.51) | 1.18 (1.01, 1.38)* |
| *Socio-Economic Indicators* |  |  |
| Receiving water through a tube well | 2.76 (1.92, 3.98) |  |
| Certified healthcare | 0.46 (0.30, 0.70) |  |
| Kacha (less solid) vs pucca house (more solid) | 1.37 (0.79, 2.39) |  |
| Household income (rupees) |  |  |
| 0-10,000 | 1 |  |
| 10,001-20,000 | 0.52 (0.33, 0.82) |  |
| >20,00 | 0.57 (0.33, 0.96) |  |
| Education level |  |  |
| None/primary | 1 |  |
| Middle/secondary | 0.53 (0.37, 0.77) |  |
| Graduate | 0.15 (0.06, 0.40) |  |
| Socio-economic status score* | 0.61 (0.53, 0.70) | 0.67 (0.57, 0.78)* |
| *Drugs (ever taken)* |  |  |
| Ever drank alcohol | 0.59 (0.39, 0.89) | 0.58 (0.36, 0.95) |
| Ever used opium/bhuki | 4.72 (2.80, 7.94) | 3.02 (1.67, 5.48) |
| Ever smoked tobacco | 1.17 (0.55, 2.50) |  |
| *Social risks* |  |  |
| Have a tattoo | 1.51 (0.89, 2.58) |  |
| Use barbers | 1.45 (0.97, 2.18) |  |
| Have body piercings | 0.84 (0.58, 1.20) |  |
| *Other variables* |  |  |
| Ever been incarcerated | 2.54 (0.97, 6.63) |  |
| Ever had a motor vehicle accident | 1.85 (1.25, 2.75) | 1.61 (1.04, 2.50) |

*For power only the combined socio-economic status score variable was included rather than the socio-economic variables, and similarly only the combined medical risk score from the individual medical risk variables. All other variables that were associated with anti-HCV in the single variable analysis were then included in the multivariable analysis.

**Reference**

1. **Sood A, et al.** The Burden of Hepatitis C Virus Infection in Punjab, India: A Population-based Serosurvey. PLoS One. 2018.
